# The association between hospital volume and overall survival in adult AML patients treated with intensive chemotherapy

**DOI:** 10.1016/j.esmoop.2025.104152

**Published:** 2025-01-30

**Authors:** Z.L.R. Kaplan, N. van Leeuwen, D. van Klaveren, F. Eijkenaar, O. Visser, E.F.M. Posthuma, S. Zweegman, G. Huls, A. van Rhenen, N.M.A. Blijlevens, J.J. Cornelissen, A.A. van de Loosdrecht, J.H.F.M. Pruijt, M.D. Levin, M. Hoogendoorn, V.E.P.P. Lemmens, H.F. Lingsma, A.G. Dinmohamed

**Affiliations:** 1 Department of Public Health, Erasmus MC, University Medical Center Rotterdam, Rotterdam, The Netherlands; 2 Department of Research and Development, Netherlands Comprehensive Cancer Organisation (IKNL), Utrecht, The Netherlands; 3 Erasmus School of Health Policy & Management, Erasmus University Rotterdam, Rotterdam, The Netherlands; 4 Department of Registration, Netherlands Comprehensive Cancer Organisation (IKNL), Utrecht, The Netherlands; 5 Department of Internal Medicine, Reinier de Graaf Hospital, Delft, The Netherlands; 6 Department of Hematology, Leiden University Medical Center, Leiden, The Netherlands; 7 Department of Hematology, Amsterdam UMC, Location VUmc, Amsterdam, The Netherlands; 8 Department of Hematology, University Medical Center Groningen, Groningen, The Netherlands; 9 Department of Hematology, Utrecht University Medical Center, Utrecht, The Netherlands; 10 Department of Hematology, Radboud University Medical Center, Nijmegen, The Netherlands; 11 Department of Hematology, Erasmus MC Cancer Institute, University Medical Center Rotterdam, Rotterdam, The Netherlands; 12 Department of Internal Medicine, Jeroen Bosch Hospital, ’s-Hertogenbosch, The Netherlands; 13 Department of Internal Medicine, Albert Schweitzer Hospital, Dordrecht, The Netherlands; 14 Department of Internal Medicine, Medical Centre Leeuwarden, Leeuwarden, The Netherlands

**Keywords:** volume–outcome association, population-based, acute myeloid leukemia, quality of care

## Abstract

**Background:**

Acute myeloid leukemia (AML) requires specialized care, particularly when administrating intensive remission induction chemotherapy (ICT). High-volume hospitals are presumed more adept at delivering this complex treatment, resulting in better overall survival (OS) rates. Despite its potential implications for quality improvement, research on the volume–outcome relationship in ICT administration for AML is scarce. This nationwide, population-based study in the Netherlands explored the volume–outcome relationship in AML.

**Materials and methods:**

Data from the Netherlands Cancer Registry on adult (≥18 years of age) ICT-treated AML patients, diagnosed between 2014 and 2018, were analyzed. Hospital volume was assessed against OS using mixed-effects Cox regression, adjusting for patient and disease characteristics (i.e. case mix), with hospital as a random effect.

**Results:**

Our study population consisted of a total of 1761 patients (57% male), with a median age of 61 years. The average annual number of ICT-treated patients varied across the 24 hospitals (range 1-56, median 13, and interquartile range 8-20 patients per hospital per year). Overall, an increase of 10 ICT-treated patients annually was associated with an 8% lower mortality risk [hazard ratio (HR) 0.92, 95% confidence interval (CI) 0.87-0.98, *P* = 0.01]. This association was not significant at 30-day (HR 1.02, 95% CI 0.89-1.17, *P* = 0.75) and 42-day (HR 0.96, 95% CI 0.85-1.08, *P* = 0.54) OS but became apparent after 100-day OS (HR 0.91, 95% CI 0.83-0.99, *P* = 0.05).

**Conclusions:**

There is a volume–outcome association within AML care. This finding could support hospital volume as a metric in AML care. However, it should be acknowledged that centralizing care is a complex process with implications for health care providers and patients. Therefore, any move toward centralization must be judiciously balanced.

## Highlights

• There is an association between hospital volume and longer term outcomes (i.e. 100-day OS).
• Further research is needed to define specific care processes and structural measures that lead to better outcomes.
• Centralization of care is a complex process with many implications for health care providers and patients.

## Introduction

Acute myeloid leukemia (AML), marked by the aggressive proliferation of abnormal white blood cells in the bone marrow, is one of the most challenging malignancies. This low-volume, high-risk malignancy requires complex diagnostic and treatment strategies, notably intensive remission induction chemotherapy (ICT) administered to eligible patients based on specific clinical and disease-related characteristics. However, ICT treatment harbors the risk of life-threatening complications such as myelosuppression, precipitating bleeding, and infections, which could ultimately lead to treatment-related early mortality (e.g. 30-day mortality after ICT initiation).^1^, ^2^, ^3^ In this high-stakes setting, balancing the risks of fatal relapse and treatment-related mortality becomes paramount. Therefore, managing these complexities requires highly experienced care providers and specialized resources.^4^ Given the potential benefits of increased practice, high-volume hospitals might be more adept in managing AML and its treatment-related sequelae.^4^^,^^5^

The volume–outcome relationship—i.e. the association between improved patient outcomes in hospitals with higher procedure frequencies—has been studied in solid malignancies.^6^ The growing evidence shows that this relationship is most pronounced in high-risk, infrequently performed surgical interventions, as seen in esophageal and pancreatic cancers.^7^, ^8^, ^9^, ^10^ This phenomenon is hypothesized to arise from two main factors: ‘practice-makes-perfect’ and ‘selective referral’, implying that higher patient volumes may enhance skills and attract more patients to better-performing hospitals.^11^, ^12^, ^13^ Consequently, hospital volume might serve as a proxy for health care processes and structure measures, potentially leading to better patient outcomes.^12^, ^13^, ^14^ As these factors are usually hard to measure and are generally challenging to convey to low-volume hospitals, centralizing care for low-volume malignancies requiring high-risk treatment is considered the primary strategy for reducing hospital variation in patient outcomes in oncological care.^6^^,^^9^^,^^15^

Despite its potential implications for quality improvement in oncological care, research on the volume–outcome relationship in non-surgical cancer treatments, especially ICT administration in AML, is notably scarce.^16^ A few studies have indicated lower early death (ED) rates in ICT-treated AML patients at academic hospitals possibly reflecting their higher patient volumes and enhanced proficiency in managing ICT-related complications.^4^^,^^5^ However, these findings are indefinite due to the scarcity of research and methodological limitations, such as inadequate adjustment for confounders and random variation.^4^^,^^5^^,^^17^, ^18^, ^19^

The complexities of AML treatment, which differ significantly from surgical interventions in solid malignancies, highlight a critical gap in the current understanding regarding the volume–outcome dynamic in hematological malignancies. To bridge this knowledge gap and deepen our understanding of the volume–outcome relationship in AML care, we conducted a nationwide, population-based study in the Netherlands to assess the association between hospital volume and patient outcomes in ICT-treated adult patients with AML.

## Materials and methods

### Data source

The data for this study were sourced from the nationwide Netherlands Cancer Registry (NCR), which has been operational since 1989 and is maintained by the Netherlands Comprehensive Cancer Organisation (IKNL).^20^ The NCR aggregates case notifications from the Nationwide Network of Histopathology and Cytopathology and the National Hospital Discharge Registry, of which the latter contains information on inpatient and outpatient discharges. Information on the date of birth and diagnosis, sex, disease stage, topography, morphology, primary treatment, and hospital of diagnosis and treatment was obtained from the medical records by trained registers of the NCR. Annually, the NCR integrates data from the Nationwide Population Registries Network for accurate updates on the patient’s vital status (i.e. alive, dead, or emigrated).

According to the Central Committee on Research Involving Human Subjects (CCMO), this observational study did not require approval from an ethics committee in the Netherlands. Approval for using anonymized data for this study was obtained from the NCR Privacy Review Board (K22.157).

### Study population

We included adults (≥18 years of age) diagnosed with AML between 1 January 2014 and 31 December 2018, from the NCR using morphology codes of the International Classification of Disease for Oncology, as described elsewhere.^21^ The start date coincides with the availability of enhanced data on prognostic factors and first-line treatment regimens. Exclusions were made for acute promyelocytic leukemia (*n* = 172), blastic plasmacytoid dendritic cell neoplasms (*n* = 37), and post-mortem diagnoses (*n* = 5).

As the effect of hospital volume is hypothesized to be associated with survival in patients who received potentially curative treatment, only those who received ICT as the primary induction treatment were included in the analyses. Patients treated with ICT outside the Netherlands were excluded from this study (*n* = 17). All patients were followed from diagnosis until death, emigration, or the last follow-up (i.e. 1 February 2022), whichever occurred first.

### Hemato-oncological care in the Netherlands

AML care in the Netherlands operates within a regional network where the diagnosis was established.^22^ Since the administration of ICT requires specialized health care providers and resources, it can only be administered in certain hospitals. Hospital eligibility for ICT administration is determined by the echelon classification system developed by the Hemato-Oncology Foundation for Adults in The Netherlands (HOVON).^23^ This system was initially established to ensure that hospitals could participate in various clinical intervention studies based on their medical facilities and structural features. Moreover, this system categorizes hospitals into five levels ranging from A to D. Level A constitutes academic hospitals qualified for all intensive hematological care procedures, including ICT administration and autologous (autoSCT) and allogeneic stem-cell transplantation (alloSCT). Level B includes hospitals that qualified for ICT and autoSCT. Level C hospitals are divided into two categories: C-HIC and C-SCT. Level C-HIC hospitals can administer ICT and provide post-SCT care, whereas level C-SCT hospitals are solely qualified for non-intensive hematological care and post-SCT care. Level D hospitals can only provide non-intensive hematological care. During our study period of 2014-2018, a total of 24 hospitals qualified to administer ICT. Notably, patients managed with ICT in a non-academic hospital who are eligible for alloSCT are referred to an academic center (i.e. level A) for subsequent treatment. Further, post-transplantation care is often provided by the hospitals that initiated ICT.

### Statistical analyses

#### Descriptive statistics

The patient and treatment characteristics of all hospitals are presented using descriptive statistics. The Kruskal–Wallis test was used to compare continuous covariates, and Fisher’s exact test was used to compare categorical covariates.

#### Hospital volume and overall survival

This study spanned 5 years (2014-2018) to examine the association between hospital volume and overall survival (OS). Hospital volume was analyzed as a continuous and categorical variable. On a continuous scale, hospital volume was defined as the total number of patients treated with ICT in a hospital within a calendar year. On a categorical scale, hospitals were divided into quarters based on the annual number of patients treated as follows: very low (quarter I, 1-8 patients per year), low (quarter II, 9-13 patients per year), medium (quarter III, 14-20 patients per year), and high (quarter IV, 21-56 patients per year).

Differences in structure and process measures were examined according to quarter volume. These included the time between diagnosis and treatment, number of ICT cycles, application of SCT, and ED rate (i.e. death within 30 days of diagnosis).

The clustering of patients in different hospitals (i.e. multilevel data) is likely to induce a correlation among patients treated in the same hospital, which demands a multilevel approach.^24^^,^^25^ Therefore, the association between hospital volume and OS was assessed using mixed-effects Cox regression. Hospital was included as a random effect and patient- and disease-specific variables (henceforth referred to as case-mix variables) as fixed effects. The case-mix variables included baseline characteristics for which prior research has shown an association with OS: age, sex, socioeconomic status (SES), secondary AML, the 2010 classification of the European LeukemiaNet (ELN), hyperleukocytosis, and participation in first-line treatment trials.^1^, ^2^, ^3^ SES is based on household income and measured on ZIP code level, and was categorized into low (decile 1-3), medium (decile 4-7), or high (decile 8-10). The ELN 2010 risk classification stratified patients into favorable, intermediate I, intermediate II, and adverse risk.^3^ Hyperleukocytosis was defined as a white blood cell count of ≥100 × 10^9^/l at diagnosis and was included as a dichotomous variable.^26^ Trial participation, defined as a dichotomous variable, was included because it could affect the OS of patients as it may act as a proxy for their health condition. After all, patients are generally enrolled in trials based on their physical and mental health state.^27^

Sensitivity analyses were carried out to assess the robustness of our primary findings. Firstly, adjustment for hospital level (i.e. academic versus non-academic) was included in our final model to assess whether the association between volume and OS could be partially explained by academic setting. Additionally, the interactions between hospital volume and age groups (≤60 years and >60 years), as well as between hospital volume and ELN risk groups, were evaluated to assess the consistency of the volume–outcome association. Secondly, as mentioned earlier, AML care in the Netherlands is organized in regional networks, wherein care provision (i.e. ICT and SCT applications and post-SCT care) is provided by multiple caregivers during the course of treatment. To gain insight into the extent to which changes in care providers influenced our results, we examined the effect of hospital volume on OS in patients who underwent all treatments (i.e. ICT and SCT) solely in an academic setting. Lastly, to investigate the fragmented structure of care and clarify the potential care processes that underlie the volume–outcome relationship, we examined the association between hospital volume at different time points after diagnosis, namely 30-day OS (i.e. after start of the first ICT cycle), 42-day OS (i.e. after two cycles of ICT), and 100-day OS (i.e. after two or three cycles of ICT, with a portion of patients having undergone SCT).

A funnel plot was created to compare the 1-year OS between hospitals in relation to the observed and expected outcomes (i.e. benchmark), taking into account statistical uncertainty. The expected 1-year mortality of each hospital was determined using Cox regression analysis, including case-mix adjustment as described previously. The 95% control limits were plotted to examine whether the 1-year mortality rate of the hospitals was significantly different from that of the benchmark. The control limits were also calculated by applying the Bonferroni correction to account for multiple testing. A detailed description of the statistical methods used for funnel plots in survival analysis is provided elsewhere.^28^

All statistical analyses were carried out using R statistical software version 1.4.1103.^29^ Random-effects modeling was conducted using the coxme package for random-effects survival modeling.^30^ *P* values <0.05 were considered statistically significant.

## Results

### Patient population

From 2014 to 2018, 4060 adults (≥18 years of age) were diagnosed with AML in the Netherlands. Among these, 1761 (43%) received ICT in 24 hospitals (Table 1). These patients had a median age at diagnosis of 61 years [interquartile range (IQR) 57-67 years], with statistically significant differences across hospitals (*P* = 0.008*)*. Also, significant between-hospital differences were observed for SES (ranging from 5% to 83% with low SES; *P* < 0.001) and ELN 2010 risk classification, of which the latter was primarily influenced by unperformed cytogenetics (ranging from 0% to 63% with no cytogenetic diagnostics carried out; *P* < 0.001). Baseline characteristics were comparable across all hospital volume quarters, except for SES, which ranged from 23% to 34% with low SES (*P* < 0.001) (Supplementary Table S1, available at https://doi.org/10.1016/j.esmoop.2025.104152).

**Table 1:** Baseline characteristics of adult (≥18 years of age) patients with AML who received treatment with ICT across 24 hospitals in the Netherlands, 2014-2018

Characteristics	Overall ICT-treated population		Range between hospitals			*P* value^a^
	*n*	(%)	Absolute number or percentage (%)			
Total no. of patients	1761	(100)	6	—	254	
Sex						0.168
Male	1012	(57)	(33)	—	(71)	
Female	749	(43)	(30)	—	(67)	
Age, years						
Median [IQR]	61 [51-67]		57 [44-64]	—	71 [68-76]	0.008
18-40	188	(11)	(0)	—	(17)	0.004
41-60	672	(38)	(0)	—	(55)	
61-70	685	(39)	(25)	—	(50)	
71-80	209	(12)	(4)	—	(50)	
80+	7	(1)	(0)	—	(4)	
Socioeconomic status						<0.001
Low	499	(28)	(5)	—	(83)	
Mid	703	(40)	(0)	—	(62)	
High	559	(32)	(8)	—	(55)	
Secondary AML	177	(10)	(0)		(16)	0.325
ELN 2010 classification						<0.001
Favorable	494	(28)	(13)	—	(71)	
Intermediate I	390	(22)	(0)	—	(50)	
Intermediate II	424	(24)	(0)	—	(33)	
Adverse	276	(16)	(0)	—	(24)	
Cytogenetic diagnostics not carried out	177	(10)	(0)	—	(63)	
Hyperleukocytosis^b^						0.349
No	1540	(87)	(71)	—	(100)	
Yes	221	(13)	(0)	—	(29)	
Trial participation	605	(34)	(0)	—	(71)	<0.001

AML, acute myeloid leukemia; ELN, European LeukemiaNet; ICT, intensive remission induction chemotherapy; IQR, interquartile range.

a *P* value is based on a comparison between 24 hospitals using the non-parametric Kruskal–Wallis test for continuous variables and Fisher’s exact test for categorical variables.

b Hyperleukocytosis is defined as a white blood cell count >100 × 10^9^/l. Two patients had missing data on white blood cell count at diagnosis.

### Differences in structure and process measures

The median time from diagnosis to the start of ICT ranged from 4 to 6 days across hospital volume quartiles. The number of ICT cycles was consistent across the volume quartiles (*P* = 0.273). Lastly, significant differences were found with the application of alloSCT across the volume quartiles (*P* = 0.035), with patients managed in low-volume hospitals proceeding to an alloSCT less frequently (34%), as compared with the remaining volume quartiles, which were similarly distributed (range 41%-45%).

### The association between hospital volume and overall survival

Over the entire study period, the total number of ICT-treated patients within a hospital ranged between 6 and 254 (Table 1). Hospital volume per year ranged from 1 to 56 patients, with a median of 13 patients (IQR 8-20 patients per hospital per year).

**Figure 1:**
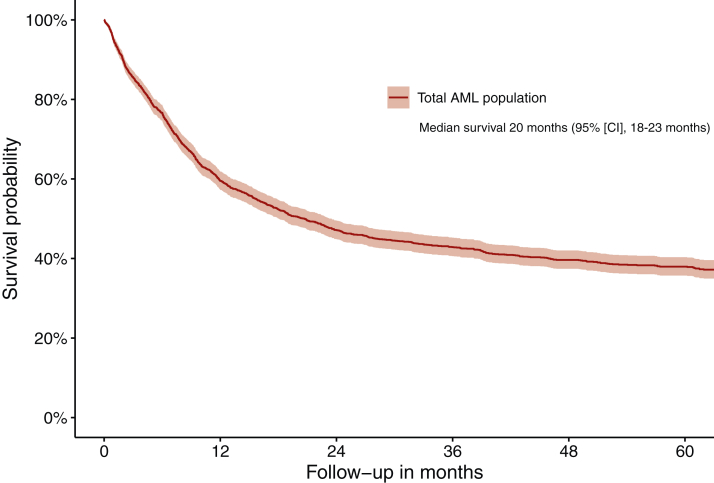
**Overall survival of patients with AML treated with ICT in the Netherlands.** This figure shows the Kaplan–Meier curve for all AML patients treated with ICT in the Netherlands between 2014 and 2018. AML, acute myeloid leukemia; ICT, intensive remission induction chemotherapy.

The unadjusted median OS for the entire study population was 20 months [95% confidence interval (CI) 18-23 months], with a 1- and 5-year OS probability of 59% (95% CI 57% to 62%) and 38% (95% CI 36% to 40%), respectively (Figure 1). Also, the unadjusted ED rates within 30 days of diagnosis showed no significant variance across hospital volume quarters (*P* = 0.781; Table 2). Of note, the median follow-up time, as calculated as per the reverse Kaplan–Meier method, was 67 months (95% CI 65-69 months).

**Table 2:** Care process measures of adult (≥18 years of age) patients with acute myeloid leukemia who received treatment with ICT across 24 hospitals in the Netherlands according to yearly hospital volume quarters, 2014-2018

Hospital volume quarters^a^						
Treatment characteristics	Total population *n* (%)	Very low *n* (%)	Low *n* (%)	Medium *n* (%)	High *n* (%)	*P* value^b^
Hospital volume per year (range)	1-56	1-8	9-13	14-20	21-56	
Days between diagnosis and start ICT, median [IQR]	5 [2-12]	5 [3-12]	4 [2-8]	4 [2-11]	6 [2-15]	<0.001
Number of ICT cycles						0.273
1	421 (24)	33 (23)	67 (22)	115 (24)	206 (24)	
2	1174 (67)	88 (62)	204 (68)	323 (68)	558 (66)	
3 or 4	166 (9)	22 (15)	28 (9)	39 (8)	76 (9)	
Stem-cell transplantation						0.035
No stem-cell transplantation	795 (45)	81 (56)	141 (47)	201 (42)	371 (44)	
Autologous stem-cell transplantation	215 (12)	14 (10)	35 (12)	71 (15)	94 (11)	
Allogeneic stem-cell transplantation	751 (43)	48 (34)	123 (41)	205 (43)	375 (45)	
Early mortality^c^	92 (5)	10 (7)	15 (5)	23 (5)	44 (5)	0.781

ICT, intensive remission induction chemotherapy; IQR, interquartile range.

a Volume quarters: very low (quarter I, 1-8 patients per year), low (quarter II, 9-13 patients per year), medium (quarter III, 14-20 patients per year), and high (quarter IV, 21-56 patients per year).

b *P* value is based on a comparison between the four volume quarters using the non-parametric Kruskal–Wallis test for continuous variables and Fisher’s exact test for categorical variables.

c Early mortality is defined as occurrence of death within 30 days after start of treatment with ICT.

**Figure 2:**
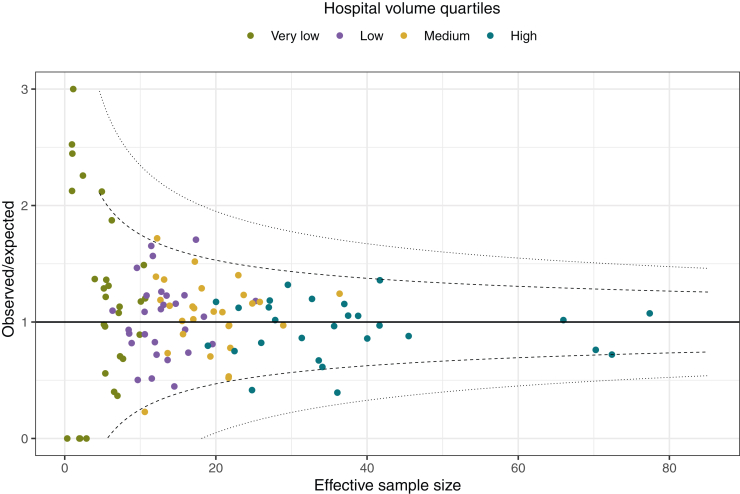
**Funnel plot for 1-year mortality following treatment with intensive chemotherapy.** This funnel plot shows each hospital for each treatment year according to hospital volume on the *x*-axis and the ratio of observed-to-expected 1-year overall survival (O/E) on the *y*-axis. The expected mortality is determined using Cox regression analyses and adjusted for case-mix variables (i.e. age, sex, socioeconomic status, secondary AML, hyperleukocytosis, and ELN 2010 risk classification). The horizontal solid line represents the value wherein the observed cases equal the expected cases. The dashed lines represent the unadjusted 95% control limits, and Bonferroni corrected control limit. The effective sample does not correspond with the hospital volume since it is defined as the hospital volume adjusted for case mix and hospital follow-up. AML, acute myeloid leukemia; ELN, European LeukemiaNet.

The funnel plot shows that the outcome of most hospitals was not significantly different from the benchmark (Figure 2). However, the funnel plot showed decreasing variability in 1-year OS between hospitals as the effective sample size increases. More specifically, very-low-volume and low-volume hospitals seem to exhibit worse outcomes (i.e. an observed-to-expected ratio >1) than higher-volume hospitals.

The multivariable mixed-effects Cox regression model shows hospital volume to be significantly associated with OS. More specifically, an increase of 10 patients treated with ICT annually within a hospital was associated with an 8% lower mortality risk [hazard ratio (HR) 0.92, 95% CI 0.87-0.98, *P* = 0.01]. This association remained significant after adjusting for hospital level (i.e. academic versus non-academic hospital) and was consistent over age groups (i.e. ≤60 years and >60 years) and ELN risk groups (Supplementary Tables S2 and S3, available at https://doi.org/10.1016/j.esmoop.2025.104152). The sensitivity analysis, which included only patients who received their treatments in academic hospitals, also showed a similar association between hospital volume and OS (HR 0.89, 95% CI 0.80-0.99, *P* = 0.04). Additionally, we observed no association between hospital volume and 30-day OS (HR 1.02, 95% CI 0.89-1.17, *P* = 0.75) and 42-day OS (HR 0.96, 95% CI 0.85-1.08, *P* = 0.54). However, a comparable association was found between hospital volume and 100-day OS (HR 0.91, 95% CI 0.83-0.99, *P* = 0.05).

## Discussion

In this nationwide, population-based study, we explored the volume–outcome association in adult AML patients treated with ICT in the Netherlands using a random-effects methodology with case-mix adjustment. Our findings indicate an association between hospital volume and OS, which became apparent after 100-day OS, contributing valuable insights to the limited literature on this subject. Additionally, we observed no association between volume and 30-day OS and 42-day OS (i.e. early mortality), which is considered to be the most difficult period of induction chemotherapy.

### Understanding the volume–outcome association

High-volume hospitals might enhance outcomes in low-frequency oncological diseases requiring complex care (e.g. surgical procedures), supporting the hypothesis that greater patient volumes might enhance health care providers’ experience and proficiency. However, hospital volume is a proxy measure reflecting underlying factors impacting care delivery, like physician expertise, organizational care processes, and multidisciplinary discussions with other hospitals and transplantation centers.^12^, ^13^, ^14^ For example, intensive care unit (ICU) admission during AML treatment is associated with high mortality, particularly in adult patients with significant comorbidity. However, a preemptive ICU admission policy—where patients are admitted before the onset of organ failure—may offer potential benefits for high-risk patients. High-volume hospitals could be better at identifying these patients early, potentially leading to improved outcomes.^31^^,^^32^ In the examination of treatment-related process measures across hospital volume quartiles, our study found limited differences. For instance, slightly higher rates of allogeneic transplantation were observed in high-volume hospitals, despite similar baseline characteristics. These findings suggest that the superiority of high-volume hospitals is related to less quantifiable factors like team dynamics, collective expertise, and institutional culture which possibly play a critical role in patient outcomes, especially in complex diseases like AML.^8^^,^^33^ Moreover, the association between hospital volume and OS persisted after adjusting for the academic nature of the center, indicating that differences in outcomes are not merely academic versus non-academic distinctions. Therefore, to develop successful quality improvement interventions, it is crucial to understand the underlying dynamics through which high-volume hospitals achieve better outcomes and to explore strategies for translating these to low-volume hospitals.^8^^,^^34^

The regional organization of AML care in the Netherlands within the HOVON network, involving multiple hospitals in a patient’s treatment journey, adds another layer of complexity in interpreting and understanding our findings on the volume–outcome relationship.^22^ This organizational structure makes it challenging to pinpoint how increased volume translates into improved outcomes and prompts the question whether the observed association should be solely attributed to the center initiating ICT.^9^^,^^22^ More importantly, when investigating the association between hospital volume and the 30-day and 42-day OS, our study reveals a uniform performance across hospital volumes during this critical early treatment period. This finding likely reflects the quality standards for personnel and facilities mandated by the HOVON echelon system for hospitals treating patients with AML.^23^

In contrast, the landscape shifts when examining OS at the 100-day mark, where a survival advantage emerges for patients initially treated at higher-volume hospitals. At that time point, most patients are after the second or even the third course of ICT, with a portion of patients having undergone autoSCT or navigating the early phases of post-allogeneic engraftment and immunosuppression. The transition of a subset of patients to other hospitals for consolidation treatment with SCT suggests that practices at the transplantation center might influence survival outcomes. After all, the conditioning regimen preceding the SCT and the transplant itself also introduce additional risks, including graft-versus-host disease and infectious complications.^1^, ^2^, ^3^ However, our focused analysis, restricted to academic centers, which have the ability to deliver full AML care within one center, showed a significant volume–outcome association. This persistent association between volume of the ICT-initiating hospital and patient survival might stem from initial treatment practices in the initiating center that influence overall health status, through mechanisms such as infection sequelae, bleeding, or compromised nutrition status.^35^ Of note, it is critical to acknowledge that not every patient requires consolidation treatment with an SCT, with some precluded by adverse events from reaching this phase of consolidation treatment.^2^ Collectively, these findings underscore once again the importance of considering the entire care continuum—from initial ICT initiation to subsequent treatments like SCT and from non-academic to academic hospital—in understanding how hospital volume impacts outcomes.

### Implications of the volume–outcome relationship

Clear volume–outcome associations have been demonstrated in studies of surgical procedures, which ultimately led to the implementation of volume thresholds, leading to significant reduction of post-operative morbidity, shorter in-hospital length of stay, and lower 30-day mortality rates.^36^^,^^37^ In light of this, our study results might also lead to the reflex of concentrating AML patients in high-volume hospitals. However, the above referenced studies do not involve the volume–outcome associations of complex care pathways involving combined modalities, longer periods of (induction) therapy, longer follow-up (i.e. beyond the 30-day mark), and involvement of multiple hospitals (e.g. transplantation center).^36^^,^^37^ Therefore, the straightforward centralization of care might have undesired effects.^38^, ^39^, ^40^, ^41^, ^42^ Access to specialized care can be compromised due to higher travel distance, potentially increasing socioeconomic inequalities, and worse outcomes for patients with lower SES.^38^^,^^39^ Furthermore, alongside good outcomes, patients also value well-functioning care pathways and processes, indicating the necessity to incorporate these in centralization processes in addition to outcomes.^41^^,^^42^ At the institutional level, centralization can lead to reduced availability of specialists and the closure of specialty programs, impacting job satisfaction and staff morale.^41^

### Strengths and limitations of the study

The main strength of our study lies in using a nationwide, population-based cancer registry (i.e. the NCR) that includes detailed data on patient and AML characteristics. The NCR offers a unique opportunity to evaluate AML care provision and evaluate the effect of hospital volume on a national scale while diminishing the chance of selection bias. Moreover, all residents of the Netherlands have equal access to high-quality health care services, irrespective of their socioeconomic position and place of residence. Therefore, treatment decision making is primarily based on patient- and disease-related factors in concert with shared decision making (i.e. including patient preferences). Another strength of our study is the use of random-effects modeling, enabling us to account for the data’s multilevel structure while accounting for differences in case mix. Unlike many studies that categorize or dichotomize hospital volume, we analyzed the effect of hospital volume on linear and categorical scales to preserve the effect size, statistical power, and reliability of our findings.^43^

Limitations of our study should also be acknowledged. As the Introduction section highlights, the volume–outcome relationship in AML care is, at least in part, based on the ‘practice-makes-perfect’ hypothesis, suggesting that high-volume centers possess more experience in conditioning patients for ICT and managing complications.^4^^,^^5^^,^^13^ However, we could not test this hypothesis directly due to the unavailability of data on these care process measures (e.g. administration of antibiotics and treatment of blood lineage dysfunctions, ICU admission) and adverse event outcomes (e.g. infections and bleeding). Incorporating registration of treatment-related processes and outcomes, such as complete remission (CR) after induction chemotherapy and time between CR and SCT, could provide deeper insights and enhance our understanding of the AML care trajectory. Lastly, during our study period, genetic risk profiling was recorded in the NCR using the ELN 2010 risk stratification. This differs from the more comprehensive and precise genotypic risk classifications of the ELN 2017 and 2022. However, these updated data are available for patients diagnosed from 2021 onward.

### Conclusion

Our study shows an association of hospital volume with longer term outcomes (i.e. 100-day OS), but not with short-term outcomes (i.e. 30- and 42-day OS) in ICT-treated adult AML patients in the Netherlands. Therefore, further research is needed to define specific care processes and structural measures that lead to better outcomes in AML treatment. Additionally, improving NCR with more comprehensive clinical and process-related data is crucial to unravel these AML-specific care processes and structure measures. Collectively, it should be acknowledged that centralizing care is a complex process with many implications for health care providers and patients. Any move toward centralization based on volume thresholds must therefore be judiciously balanced. This balance should account for potential disadvantages and be informed by a collaborative, evidence-based approach, ensuring that any policy decisions made are in the best interest of patients and the overall health care system.
