# Supplementary material for: The association between hospital volume and overall survival in adult AML patients treated with intensive chemotherapy
Source: ESMO Open. 2025 Jan 30;10(2):104152. doi: 10.1016/j.esmoop.2025.104152 (PMC11833631; doi:10.1016/j.esmoop.2025.104152)
Supplement: Supplemental material [file mmc1.docx]

# Supplemental material

# Title

The association between hospital volume and overall survival in adult AML patients treated with intensive chemotherapy

**Running title**

The volume-outcome relationship in AML

**Supplemental tables**

**Supplemental Table 1.** Baseline characteristics of adult (≥18 years) patients with acute myeloid leukemia who received treatment with intensive remission induction chemotherapy across 24 hospitals in the Netherlands, according to yearly hospital volume quarters, 2014-2018

| **Characteristics** | **Hospital volume quarters*** | | | | ***P*-value**** |
| --- | --- | --- | --- | --- | --- |
|  | **Very low**  No. (%) | **Low**  No. (%) | **Medium**  No. (%) | **High**  No. (%) |  |
| **Sex** |  |  |  |  | 0.102 |
| Male | 69 (48) | 168 (56) | 277 (58) | 498 (59) |  |
| Female | 74 (52) | 131 (44) | 200 (42) | 344 (41) |  |
| **Age, years** |  |  |  |  |  |
| Median [IQR] | 60 [51-66] | 62 [51-68] | 60 [50-67] | 61 [52-68] | 0.445 |
| 18-40 | 19 (13) | 26 (9) | 56 (12) | 87 (10) | 0.747 |
| 41-60 | 54 (38) | 110 (37) | 190 (40) | 318 (38) |  |
| 61-70 | 53 (37) | 127 (43) | 167 (35) | 338 (40) |  |
| 71-80 | 16 (11) | 35 (12) | 61 (12) | 97 (12) |  |
| 80+ | 1 (<1) | 1 (<1) | 3 (1) | 2 (<1) |  |
| **Socioeconomic status** |  |  |  |  | 0.002 |
| Low | 49 (34) | 76 (25) | 108 (23) | 266 (32) |  |
| Mid | 47 (33) | 113 (38) | 210 (44) | 333 (40) |  |
| High | 47 (33) | 110 (37) | 159 (33) | 243 (29) |  |
| **Secondary AML** | 11 (8) | 28 (9) | 39 (8) | 99 (12) | 0.133 |
| **ELN 2010 classification** |  |  |  |  | 0.229 |
| Favorable | 47 (33) | 78 (26) | 138 (29) | 231 (27) |  |
| Intermediair-I | 29 (20) | 69 (23) | 115 (24) | 177 (21) |  |
| Intermediair-II | 30 (21) | 70 (23) | 109 (23) | 215 (26) |  |
| Adverse | 17 (12) | 42 (14) | 75 (16) | 142 (17) |  |
| Cytogenetic diagnostics  not performed | 20 (14) | 40 (13) | 40 (8) | 77 (9) |  |
| **Hyperleukocytosis**^†^ |  |  |  |  | 0.522 |
| No | 130 (91) | 260 (87) | 420 (88) | 728 (87) |  |
| Yes | 13 (9) | 39 (13) | 57 (12) | 112 (13) |  |
| **Trial participation** | 48 (34) | 103 (34) | 174 (37) | 281 (33) | 0.717 |

Abbreviations: IQR, interquartile range; AML, acute myeloid leukemia; ELN, European LeukemiaNet.

^†^Hyperleukocytosis is defined as a white blood cell count greater than 100x10^9^/L. Two patients had missing data on white blood cell count at diagnosis.

*Volume quarters: very low (quarter I, 1-8 patients per year), low (quarter II, 9-13 patients per year), medium (quarter III, 14-20 patients per year), and high (quarter IV, 21-56 patients per year)

***P*-value is based on a comparison between the 4 volume quarters using the non-parametric Kruskal Wallis test for continuous variables and the Fishers`s exact test for categorical variables

**Supplemental Table 2.** Hazard rates of hospital volume for age groups ≤60 years and >60 years. P-value for likelihood ratio test comparing model with and without interaction term was 0.19.

|  | **Hazard ratio [95% CI]** |
| --- | --- |
| **Hospital volume: ≤60 years** | 0.90 [0.84-0.97] |
| **Hospital volume: >60 years** | 0.93 [0.87-1.00] |

**Supplemental Table 3.** Hazard rates of hospital volume for ELN 2010- risk groups. P-value for likelihood ratio test comparing model with and without interaction term was 0.13.

|  | **Hazard ratio [95% CI]** |
| --- | --- |
| **Hospital volume: Favorable** | 1.00 [0.91-1.00] |
| **Hospital volume: Intermediate I** | 0.91 [0.82-1.02] |
| **Hospital volume: Intermediate II** | 0.94 [0.85-1.04] |
| **Hospital volume: Adverse** | 0.87 [0.78-0.96] |
| **Hospital volume: No cytogenetics performed** | 0.84 [0.71-0.99] |
